# Bibliographical Notices

**Published:** 1844-02-24

**Authors:** 


### BIBLIOGRAPHICAL NOTICES.

*Statistical Report of One Hundred and Ninety Cases of Insanity, admitted into the Retreat, near Leeds, (England,) during ten years, from* 1830 *to* 1840. By Samuel Hare.

This is a highly interesting report, abounding in details, methodically arranged, on all the points relating to the the subject of insanity. Mr. Hare very properly remarks that "facts constitute the only basis on which the study of this, as of other diseases, can be founded;" and it is from statistical reports; carefully prepared like the present, that we must derive that useful knowledge. The report contains twenty-five *Tables*, interspersed with many judicious comments and explanations in reference to the bearing of the facts observed on the important objects of inquiry.

---

*Fifth Annual Report of the Directors and Superintendent of the Ohio Lunatic Asylum, &c.* Dec. 13, 1843.

William M. Awl, M. D., is the Superintendent, and the present report speaks well of his competency.

Beside fiscal matters, some accounts of individual cases, and other things of less interest, this report contains a very satisfactory statement, in tabular form, of the number of admissions, deaths, causes of insanity, &c. &c.

The following "recapitulation" exhibits the actual working of the Institution, as seen in the results of cases.

| | | |
|---|---|---|
| Whole number of patients admitted in five years, | | 473 |
| Males, | 248 | |
| Females, | 225—473 | |
| Old cases, | 259 | |
| Recent do. (*less duration than one year*) | 214—473 | |
| Paupers, | 349 | |
| Pay patients, | 124—473 | |
| Single, | 226 | |
| Married, | 203 | |
| Widows, | 33 | |
| Widowers, | 11—473 | |
| Whole number discharged | Recovered, | 203 |
| | Improved, | 18 |
| | Incurable, | 51 |
| | Idiotic, | 2 |
| | Died, | 51—325 |
| Whole number of recent cases discharged, | Recovered | 154 |
| | Incurable, | 4 |
| | Died, | 17—175 |
| Whole number of old cases discharged, | Recovered, | 49 |
| | Incurable, | 67 |
| | Died, | 34-150-325 |
| Per cent. of recoveries on all cases admitted in 5 years, | | 42.91 |
| Per cent. of recoveries on all cases discharged in 5 years, | | 62.46 |
| Per cent. of recoveries on all recent cases discharged in 5 years, | | 88.00 |
| Per cent. of recoveries on all old cases discharged in 5 years, | | 32.66 |
| Per cent. of deaths on the whole number, (51 of 473) | | 10.70 |
| Average per cent. of deaths in 5 years, | | 8.65 |

It is cheering to the heart of the philanthropist to observe the successful operation of these various enterprises for the relief of those laboring under that greatest of all deprivations—the loss of reason. In England and in different parts of the United States, the most laudable efforts are made for this purpose. Much, however, remains to be done. When we consider the hopeless and abandoned condition of these afflicted members of the human family, a few years since, in contrast with the comfortable provision which now obtains for such in many places, and the very large proportion who are daily restored to Society and their families, by judicious management, we can only marvel that any Christian community can be laggard in a matter of such magnitude. In and about Philadelphia, for the most part, the insane are properly cared for; but alas, for other parts of Pennsylvania,—for the *State!* A few years since, some of our spirited and benevolent citizens united in efforts to effect the establishment of a State Institution. A law was obtained for the purpose, but, unfortunately, *politics* and the unholy spirit of mammon were infused into it, and its friends were forced to abandon the project until purer patriotism and better counsels shall prevail.

---

*Human Physiology, with upwards of three hundred illustrations.* By Robley Dunglison, M.D., Professor of the Institutes of Medicine, etc., in Jefferson Medical College, Philadelphia; Attending Physician and Lecturer on Clinical Medicine at the Philadelphia Hospital; Secretary to the American Philosophical Society, etc. etc. Fifth Edition. *Greatly modified and improved.* In two volumes, Octavo. Philadelphia: Lea and Blanchard, 1844.

There are few authors in our profession whose productions have been so favourably received as those of Professor Dunglison. Scarcely a year passes without some new work, or new Edition of one already published, from the prolific pen of this accomplished writer. The secret of this great success, consists in the judicious selection and arrangement of his matter, his remarkable perspicuity of expression, and the care and industry displayed in posting up the latest facts and views of authoritative writers on the subjects of which he treats. This new Edition of "Human Physiology," exemplifies the correctness of these remarks. All the acknowledged facts recently discovered, tending to elucidate the principles of this important science, are brought up to the day. In doing so, he has necessarily been obliged to refer to the numerous works published on the subject, and to compare and comment upon the views of such distinguished men as Wagner, Gerber, Liebig, &c. This, however, is done with so much fairness to the authors and his readers, as to commend his remarks to the confidence of all candid minds.

"To this edition nearly ninety wood-cuts have been added, to elucidate either topics that had been already treated of in the previous Edition, or such as are new in this; most of the old cuts have been re-touched; and many replaced by others that are superior." The work is well printed, and on good paper—the wood-cuts are much superior to those in former editions, and altogether, it is "well got up."

